# Oxygen and mechanical ventilation impede the functional properties of resident lung mesenchymal stromal cells

**DOI:** 10.1371/journal.pone.0229521

**Published:** 2020-03-06

**Authors:** Alvaro G. Moreira, Sartaj K. Siddiqui, Rolando Macias, Teresa L. Johnson-Pais, Desiree Wilson, Jonathon A. L. Gelfond, Margarita M. Vasquez, Steven R. Seidner, Shamimunisa B. Mustafa

**Affiliations:** 1 Division of Neonatology, Department of Pediatrics, University of Texas Health Science Center San Antonio, San Antonio, Texas, United States of America; 2 Department of Urology, University of Texas Health Science Center San Antonio, San Antonio, Texas, United States of America; 3 Department of Periodontics, University of Texas Health Science Center San Antonio, San Antonio, Texas, United States of America; 4 Department of Epidemiology and Biostatistics, University of Texas Health Science Center San Antonio, San Antonio, Texas, United States of America; University of Alabama at Birmingham, UNITED STATES

## Abstract

Resident/endogenous mesenchymal stromal cells function to promote the normal development, growth, and repair of tissues. Following premature birth, the effects of routine neonatal care (e.g. oxygen support and mechanical ventilation) on the biological properties of lung endogenous mesenchymal stromal cells is (L-MSCs) is poorly understood. New Zealand white preterm rabbits were randomized into the following groups: (i) sacrificed at birth (Fetal), (ii) spontaneously breathing with 50% O_2_ for 4 hours (SB), or (iii) mechanical ventilation with 50% O_2_ for 4h (MV). At time of necropsy, L-MSCs were isolated, characterized, and compared. L-MSCs isolated from the MV group had decreased differentiation capacity, ability to form stem cell colonies, and expressed less vascular endothelial growth factor mRNA. Compared to Fetal L-MSCs, 98 and 458 genes were differentially expressed in the L-MSCs derived from the SB and MV groups, respectively. Gene ontology analysis revealed these genes were involved in key regulatory processes including cell cycle, cell division, and angiogenesis. Furthermore, the L-MSCs from the SB and MV groups had smaller mitochondria, nuclear changes, and distended endoplasmic reticula. Short-term hyperoxia/mechanical ventilation after birth alters the biological properties of L-MSCs and stimulates genomic changes that may impact their reparative potential.

## Introduction

Every year upwards of 15,000 premature neonates are diagnosed with a devastating lung disease, known as bronchopulmonary dysplasia, (BPD) [1]. BPD is a multifaceted disease, attributed to prolonged exposure to supplemental oxygen and mechanical ventilation [2]. Other contributing factors to the development of BPD include inflammation, maternal health, poor nutrition, and a genetic/epigenetic predisposition to the disease. [3]. Hallmarks of BPD include an interruption of normal alveolar development, decreased pulmonary vascular growth, and impaired lung function [4]. Infants diagnosed with BPD are at increased risk for developing asthma, early-onset emphysema, and life-long cardiopulmonary disease [4]. Current management options of BPD such as gentle ventilation, antenatal steroids, postnatal surfactant, caffeine, and vitamin A are designed to not only increase survival of the preterm infant but also lessen lung injury [5]. Unfortunately, most therapeutic options have not improved the incidence of BPD.

Throughout life, the lungs are continuously exposed to damaging stimuli such as aeroallergens, pollutants, changes in oxygen tension, and pathogens. Maintaining homeostasis requires an ongoing balance of repair/regenerative processes. Among the most important cell populations regulating these repair mechanisms are resident/endogenous lung mesenchymal stromal cells (L-MSCs) [6,7]. Characteristically, MSCs are multipotent cells capable of: (i) giving rise to numerous cell types, (ii) secreting biologic factors necessary for growth and development, and (iii) supporting tissue integrity during normal cell turnover and injurious episodes [8].

Preterm neonates experience bouts of hyperoxia, inflammation, and ventilator-induced lung injury. Recent animal studies by Collins *et al* demonstrated that the repair potential of L-MSCs isolated from a rat model of hyperoxia-induced BPD was altered [9]. Furthermore, studies by Popova and colleagues suggest that the presence of MSCs in tracheal aspirates of preterm newborns was a predictor for developing BPD [10]. When cultured, these MSCs exhibited a myofibroblast phenotype suggesting these cells exhibit a dual mode of action (reparative vs. damaging) that is dependent upon their environment. Specifically, under normal circumstances L-MSCs promote lung growth and development, whereas under constant injury the cells may very well switch to a pathogenic pathway [11,12].

To better delineate the effect the microenvironment exerts on the biological properties of lung tissue resident/endogenous mesenchymal stromal cells, we tested the hypothesis that the L-MSCs isolated from preterm rabbits exposed to brief periods of supplemental oxygen or mechanical ventilation with elevated levels of oxygen would decrease their biological properties. We used this animal model for the following reasons: (i) they recapitulate the histologic and physiologic findings characteristic of human BPD, (ii) preterm kits are able to survive, (iii) rabbits are a cost-effective, large animal model, and (iv) despite being preterm, mechanical ventilation is more feasible given their size [13].

## Materials and methods

### Animals and experimental procedure

The animal protocol was reviewed and approved by the Animal Care and Use Committee of the University of Texas Health Science Center at San Antonio. Animals were maintained and cared for in an American Association for Accreditation Laboratory Animal-accredited facility. Four time-dated pregnant New Zealand white rabbits were purchased from Charles River Laboratories (Wilmington, MA). Fetuses (kits) were delivered following caesarean section at 29day gestational age (GA, term = 31days). 29day GA preterm rabbits can survive for a few hours in an incubator supplemented with 50% O_2_ (hyperoxia) with or without ventilator support [14,15]. Kits were randomized to one of the following groups: sacrificed at birth (Fetal, n = 7), spontaneously breathing, 50% O_2_ for 4h (SB, n = 12), or mechanically ventilated with 50% O_2_ for 4h (MV, n = 9). The average birthweight (mean ± SEM) for the Fetal, SB, and MV animals was 30. 3g ± 3.2g, 31.1g ± 2.0g, and 37.9g ± 1.8g, respectively.

The preterm rabbit was utilized as our experimental animal model, as at full-term gestation (31 days) the pattern of lung development is comparable to that observed in humans unlike postnatal maturation of developing lungs in rats and mice [14]. In this study, day 29 of gestational age (GA) was chosen as representative of the terminal saccular stage of rabbit lung development. Furthermore, 29-day GA preterm rabbits express limited quantities of surfactant and can only survive for short periods of time in a heated incubator supplemented with 50% O_2_ [15].

Kits assigned to the SB group were gently placed in an incubator at 37°C saturated with 50% O_2_. Kits assigned to the MV group were anesthetized with a standard rabbit cocktail consisting of xylazine (100 mg/ml, cat # 033198, ketamine (100 mg/ml, cat # 0555853), and acepromazine (10 mg/ml, cat # 003845) at a dose of 1mL/1.5kg given intramuscularly prior to tracheotomy placement (Henry Schein, Dublin, OH). Animals were subsequently transferred to individual temperature-controlled plethysmographs operated by a time-cycled, pressure-limited ventilator; using 50% O_2_ and a fixed rate of 30 breaths/min. Peak inspiratory pressures were individually regulated to maintain tidal volume at 10 ml/kg as measured with a pneumotachometer [15]. Animals were continuously monitored throughout the duration of the experiments. Kits in the SB and MV groups were necropsied at 4h of life. One animal in the SB group and two in the MV group died before the 4-hour experimental endpoint. All surviving animals were included in the experimental analysis. The adult rabbit was euthanized with an intracardiac delivery of Euthasol (100mg/kg, Henry Schein, Dublin, OH). Rabbit kits were sacrificed via intraperitoneal injection of Euthasol (0.22ml/kg) followed by thoracotomy. Whole lung tissue was excised from the thoracic cavity, rinsed in an anticoagulant citrate phosphate dextrose adenine buffer diluted with PBS (CPDA-1, Sigma-Aldrich, St. Louis, MO), to remove extraneous blood, and then placed in the same buffer at room temperature for subsequent isolation of L-MSCs.

### Isolation of L-MSCs

MSCs were isolated from whole lung tissue as per a previously described methodology [9]. Briefly, residual large airways were removed and discarded. Tissue was then minced into small pieces followed by enzymatic digestion (30U Neutral Protease, 2500U Collagenase I and 500U DNase (Sigma-Aldrich, St. Louis, MO) with gentle agitation for 60min at 37°C. The heterogeneous cell suspension was then layered on 3mL Ficoll-Paque (1.073 g/cm3, Stemcell Technologies, Cambridge, MA). Following density gradient centrifugation (20 min, 900rcf, 5/10 acceleration, 0/10 deceleration), cells accumulating in interphase were collected, washed with PBS and resuspended in α-MEM media (Sigma-Aldrich) supplemented with 20% fetal bovine serum (FBS, Sigma-Aldrich), 4mM L-glutamine and 1% penicillin/streptomycin (Thermo Fisher, Waltham, MA). Cells were counted and then cultured in plastic tissue culture flasks at 37°C in 21% O_2_. After 48 hours, flasks were rinsed with media (to remove non-adherent cells) and expanded by replacing new media every 3–4 days until 80% confluency. Cells were then passaged and plated at a density of 1 X 10^4^ cells/cm^2^ for experimental use. All experiments utilized cells from passages 1–3 and each experimental measurements from all groups were concurrently performed.

### Flow cytometry analysis of cell surface markers

Lung cells from each representative group were used for surface antigen phenotyping. Fluorescein isothiocyanate (FITC), phyco-erythrin (PE), Allophycocyanin (APC) or Alexa Fluor®647-coupled antibodies against: (i) immune complex markers CD11b (BioLegend, San Diego, CA, cat#1011220) and CD79a (GeneTex, Irvine, CA, cat#GTX80093); (ii) hematopoetic markers CD117 (BD Biosciences, San Jose, CA, cat#56118) and HLA-DR (BD Biosciences, cat#559868); and (iii) MSC-positive markers CD44 (Thermo Fisher, cat#ab5519) and CD81 (BD Biosciences, cat#561956) were utilized for flow cytometric analysis following the manufacturer’s instructions. CD44 and CD81 are previously reported MSC markers specific to rabbits [16–19]. Binding of antibodies against the markers was detected by anti-mouse immunoglobulin (IgG) conjugate, isotype-specific FITC or PE conjugated goat anti-mouse IgG (Becton Dickinson, Franklin Lakes, NJ). Confluent cells were trypsinized, washed with phenol-red free α-MEM media, aliquoted, and resuspended at a concentration of 1million cells/ml. Cells were incubated for 30 min with either conjugated specific antibodies or isotype-matched control mouse IgG at recommended concentrations at 4°C. Labeled cells were washed, resuspended in PBS, and analyzed on a Beckton Dickinson LSR II Flow Cytometer. Using forward and side scatter profile; we used doublet discrimination gating to remove debris and doublets. Dead cells were gated out using 7AAD staining. Results are expressed as the mean percentage of positive cells.

### Colony Forming Unit Efficiency (CFU-E)

L-MSCs were plated at a density of 150 cells in a 60-mm culture dish and CFU was conducted according to our previous work [20]. The colony-forming efficiency of cells (expressed as %) was calculated by dividing the number of colonies per dish by the number of cells seeded per dish (150) x 100. The CFU-E assay was performed in triplicate with three separate cell samples from each group.

### Lineage differentiation of L-MSCs

L-MSCs were stimulated *in vitro* towards adipogenic, osteogenic and chondrogenic lineages as previously described [20]. Cells were plated in T-75 flasks and once confluent, were trypsinized, counted, and plated for the specific differentiation lineage experiment. Following completion of each lineage differentiation, an Olympus CKX41 Inverted Microscope with Cellsense Imaging (Center Valley, PA) software was used for visualization and subsequent imaging.

### Migration assay

L-MSCs were seeded in 12-well culture plates at a density of 4 x 10^4^ cells/well and grown to confluency. Wells were pre-marked with reference points for imaging. The cell monolayer was rinsed with PBS and scratched using a sterile 200μl pipette tip. The cells were washed again with PBS to remove any dislodged cells before cell media was added to the well. Cell migration was measured over a period of 24h using a phase contrast microscope (Olympus IX50) equipped with the Easy Grader V3.0 imaging software. The reference point of the dish was outside of the capture image but within the eye piece field of view to capture the 0h time point. Cells were incubated for 24h, acquiring images every 2h for the first 10h and then at 24h [20]. The remaining scratch area was measured using ImageJ software. The percentage closure was calculated by using the cell surface area at a specific time point divided by the total surface area of the scratch at time 0h x 100.

### Proliferation assay

Cell proliferation was evaluated using the colorimetric AlamarBlue assay (Thermo Fisher, Waltham, MA). L-MSCs isolated from each experimental group were seeded in 96-well cell culture plates at a cell density of 7.5 x 10^4^ cells/ml in a total volume of 250 μl. As a negative control, AlamarBlue reagent was added to cell media in the absence of cells. Cells were allowed to adhere for 4h at 37°C before 25μL of the AlamarBlue reagent was added to each well. Culture plates were then wrapped in aluminum foil to protect from light and returned to the incubator. Absorbance readings at 570 and 600 nm were obtained using a spectrophotometer (BioTek, Winooski, VT) at different time points (0, 2, 4, 6, 8, 10, 12, 24, 48, 72, 96, 120 and 144h). Three independent experiments were performed, with quadruplicate samples for each group of L-MSCs. The percent reduction of AlamarBlue for each sample was calculated according to the manufacturer’s instructions.

### Transmission Electron Microscopy (TEM)

To assess morphological alterations in L-MSCs associated with postnatal adaptation, L-MSCs (1x10^6^/mL) isolated from each experimental group were resuspended in 1mL of fixative reagent (4% phosphate buffer, 1% formaldehyde, 2.5% glutaraldehyde, Sigma-Aldrich). Cells were then rinsed with 0.1M phosphate buffer for 5 mins and then placed in 1% Zetterqvist’s buffered osmium tetroxide for 30mins (Sigma-Aldrich). The samples were then dehydrated and finally placed in resin. Following this, cells were imaged using a JEOL 1230 microscope at 8K and 25K resolution by the Electron Microscopy Core Facility, University of Texas Health Science Center. Photographs were then reviewed by a pathologist blinded to the different experimental groups. To assess dilation of the rough endoplasmic reticuli (rER), 4–8 rER were randomly picked by the pathologist within each cell with 3–4 cells reviewed in each group. The organelle length was drawn longitudinally and the center (midpoint) of the length was used as the reference point to measure the width of the rER, indicative of the relative amount of dilation. Measurements are reported as μm.

### Western blot analysis

L-MSCs were homogenized in lysis buffered (Tris-HCl 50 mM, pH 7.4, TBS) supplemented with Mini Tablet Protease Inhibitor Cocktail (Sigma-Aldrich) and whole cell lysate protein concentration was determined using a Nanodrop. 30μg of total protein was subjected to SDS-PAGE. The separated proteins were transferred electrophoretically to PVDF membranes (Millipore, Bedford, MA) using a semi-dry transfer blot system and blocked in TBS containing 5% non-fat dried milk powder for 1h and then incubated with the SOX-2 primary antibody overnight. The blots were then incubated with secondary antibody conjugated to horseradish peroxidase in the same buffer for 1h. Peroxidase-labeled proteins were visualized using an enhanced chemiluminescence (ECL) assay kit (Thermo Fisher). Blots were re-probed for β-actin (Sigma-Aldrich) to control for equal loading of samples. The relative intensities of the bands were quantified by densitometry using the NIH Image J software.

### RNA isolation and microarray procedure

Total RNA was extracted from 90–100% confluent L-MSCs primary culture using the TRIzol (Invitrogen, Carlsbad, CA) reagent according to the manufacturer’s instructions. RNA was quantified on a Nanodrop ND-200 spectrophotometer (Thermo Fisher). The quality was assessed using the Agilent 2200 TapeStation Bioanalyzer (Agilent Technologies, Santa Clara, CA). The microarray gene expression procedure was performed by the institutional Genomic Core Facility, UTHSCSA. Briefly, cyanine 3-labeled cRNA probes were created from 100–200 ng of RNA using the Low Input Quick Amp Labeling kit (Agilent Technologies) according to the manufacturer’s directions. The cRNA was then hybridized to the Agilent G3 Rabbit GE 4x44K Microarray Kit (43,803 rabbit probes represented, Agilent Technologies) at 65°C for 17 h in a shaking incubator. The arrays were washed at 37°C for 1min. Dried arrays were then scanned using an Agilent SureScan microarray scanner to assess the fluorescent signals. Agilent Feature Extraction program was used to further analyze the scanned images from the arrays. The feature-extracted data was then imported into the GeneSpring GX software, version 9 (Agilent Technologies) for statistical analysis of the expressed probe IDs to identify the genes and pathways that were differentially expressed.

### Pathway analysis

Differentially expressed genes from the microarray study were further analyzed using the software application Ingenuity Pathway Analysis (IPA, Ingenuity Systems, Redwood City, CA) to identify networks associated with regulators, signaling pathways, or biological functions.

### RT-PCR

Total RNA was isolated from confluent L-MSCs as described above. cDNA was synthesized using the High Capacity cDNA Reverse Transcription kit (Applied Biosystems, Foster City, CA). Gene expression levels were analyzed using the qRT-PCR method. Rabbit-specific primer-probe sets were either commercially acquired or TaqMan Gene Expression assays were designed by submitting the cDNA sequence into Applied Biosystems custom design tool software. (TaqMan Gene Expression Assays, Applied Biosystems, Thermo Fisher). The sequence of primers used are shown in S1 Table, (S1). Relative RNA expression was normalized to GAPDH.

### Statistical analysis

All data are presented as group means ± SEM. All experiments were performed independently three-five times in triplicate and n indicates the number of independent cell populations isolated from individual kits. Analysis of variance was used for multiple group comparisons when data was normally distributed. Statistically significant results were followed for intergroup differences using Tukey’s post hoc with SPSS 24.0 software (SPSS Inc., Chicago, IL). Kruskall-Wallis was used for multiple group comparisons of nonparametric data. Dunn’s test of multiple comparisons followed a significant Kruskall-Wallis test. A p-value of <0.05 was considered statistically significant.

Microarray data was analyzed with GeneSpring, R software, and Ingenuity pathway analysis (IPA). Differentially expressed genes between groups was identified by a 1.5-fold change. RT-PCR data is represented as 3 independent experiments, bar graphs represent the mean ± SEM. *p < 0.05. Results analyzed using a one tail t-test with a p-value < 0.05 between comparison groups.

## Results

### Effect of hyperoxia and mechanical ventilation on rabbit L-MSCs

**Fig 1:**
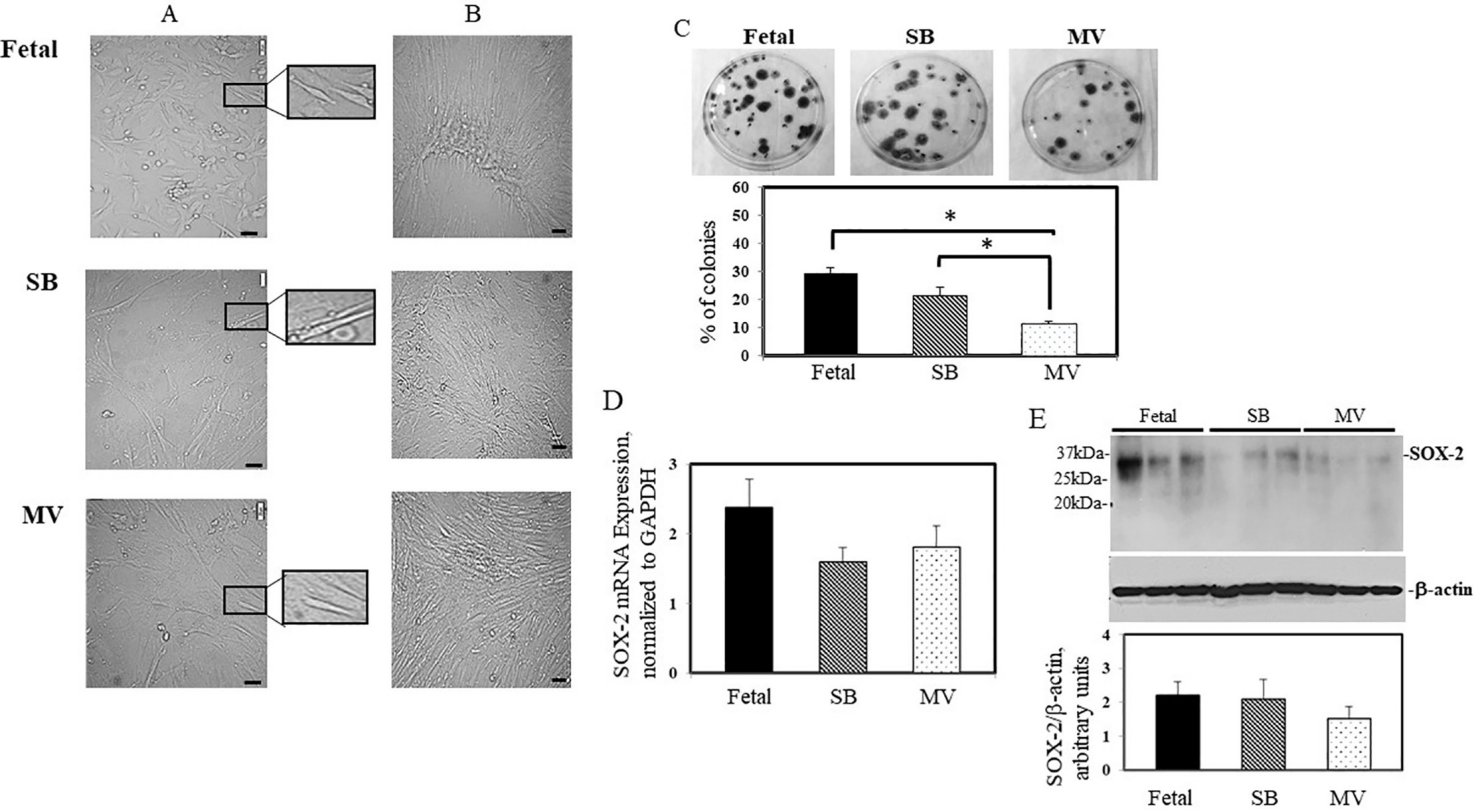
Morphology, colony-forming capacity and SOX-2 expression of endogenous preterm rabbit lung mesenchymal stromal cells (L-MSCs). Cells were cultured under normoxic conditions from each of our three groups: sacrificed at birth (Fetal), spontaneously breathing, 50% O_2_ for 4h (SB), and mechanically ventilated with 50% O_2_ for 4h (MV). Panel A; 2–4 days in culture and Panel B; 5–7 days in culture. Phase contrast images depicts cells as flat, elongated, spindle shaped monolayers. Representative area is enlarged to the right of Panel A. Black scale bars = 100μm. Panel C, Colony-forming capacity of L-MSCs, mean values of 3 separate experiments ± SEM. *, *P<0.05, Fetal vs MV and SB vs. MV. Panel D, following isolation of total RNA from cultured L-MSCs, SOX-2 mRNA expression was assessed by real-time RT-PCR and then normalized to GAPDH mRNA levels. The results are the mean ± S.E of three separate experiments performed in triplicate. Panel E, whole cell lysates were subjected to Western blot analysis. A representative image is depicted. Graphical densitometric analysis normalized to β-actin is also shown as the mean ± S.E.

Representative images of L-MSCs isolated from each group exhibited characteristic fibroblast-like, elongated, spindle-shaped morphology over a period of 7days in culture (Fig 1A; 2–4 days in culture and 1B; 5–7 days in culture and the enlarged images to the right of panel A). Cultured lung cells formed distinct colonies when plated at low density (as depicted in Fig 1C). Lung cells isolated from kits exposed to MV with 50% O_2_ showed a significant decrease in the number of colonies compared to the Fetal and SB groups. Transcription factors such as SOX-2 and NANOG regulate proliferation and totipotency in embryonic stem cells; it has been proposed that these factors play a similar role in multipotent mesenchymal stromal cells [21]. To this end, expression of these factors was evaluated in L-MSCs. Expression of NANOG mRNA by RT-PCR analysis was undetectable in L-MCSs isolated from each of our experimental groups. SOX-2 mRNA was identified in L-MCSs isolated from each of our experimental groups although mRNA levels expressed in L-MSCs from SB and MV kits were decreased compared to expression in L-MSCs from Fetal kits, but did not reach statistical significance (Fig 1, panel D). No significant differences were observed in SOX-2 protein levels in L-MSCs isolated from each experimental group as evidenced by immunoblotting analysis (Fig 1, panel E).

Flow cytometry analysis revealed a positive response to stem cell markers CD81 and CD44 surface antigens and negative staining for the hematopoietic and immune cell markers, for all three experimental groups (Table 1). A decrease in CD81 expression in L-MSCs isolated from the MV experimental group was detected when compared to lung cells from the SB group.

**Table 1:** Flow cytometric analysis of lung cells. Flow cytometry results for each cohort of L-MSCS show high similarity for surface antigens CD44 and CD81. L-MSCs were negative for immune cell marker CD11b, and hematopoietic markers CD79a, CD117 and HLA-DR. Values are presented as the mean + SEM for surface antigen marker. n = 4–6 for each group.

	Fetal	SB	MV
CD44	93±1.0	94±0.8	94±0.2
CD81	93±1.7	98±0.6	88±3.0*
Negative Markers (CD11b, CD79a, CD117, HLA-DR)	7±1.4	5.7±1.4	11±1.6

Mean ±SEM, %

*P<0.05, SB vs MV.

**Fig 2:**
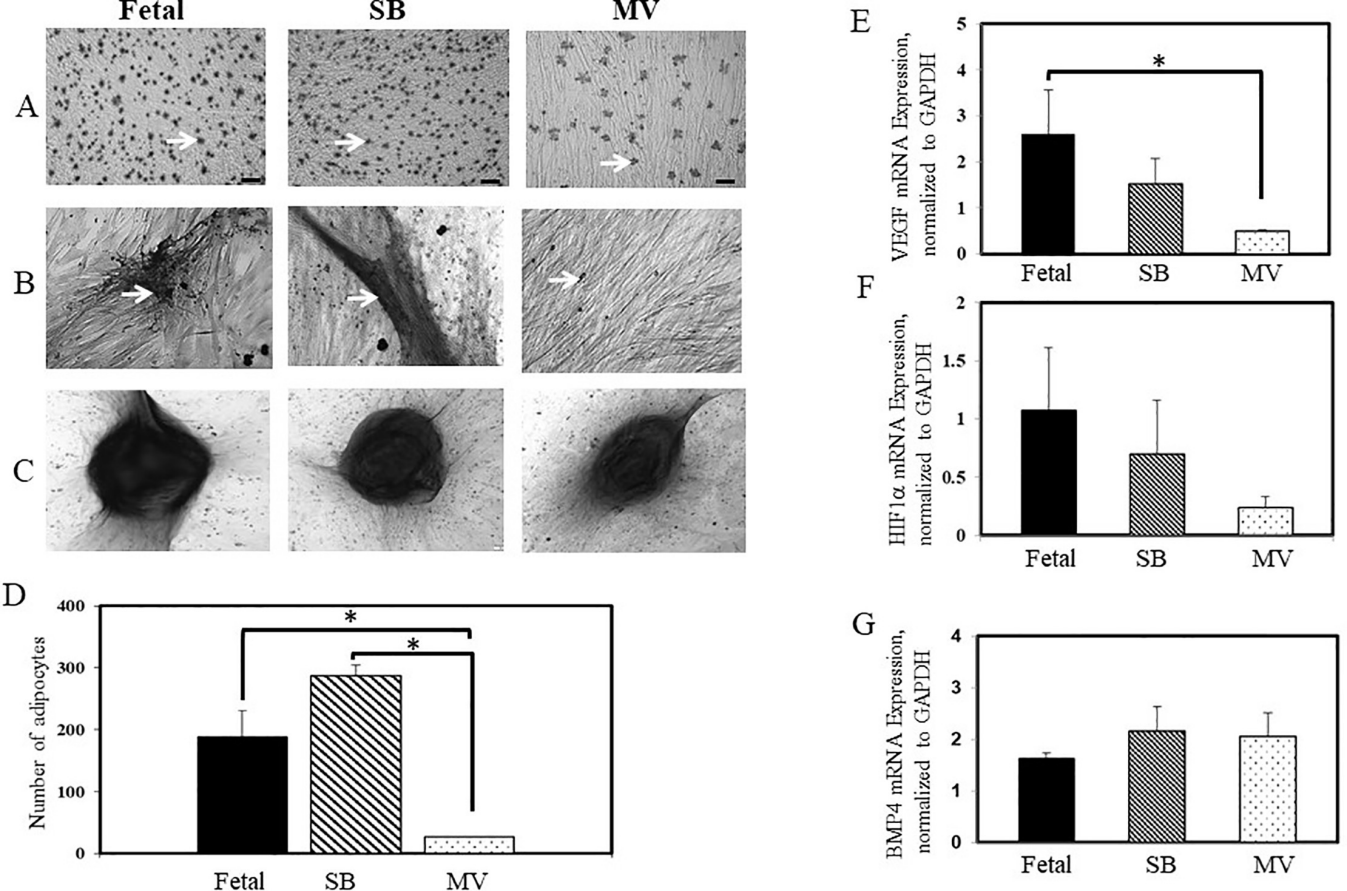
Trilineage differentiation of L-MSCs and expression of growth factors. Panel A, cells stained with oil red-O following 21 days in adipogenic culture media. Panel B, cells stained with Alizarin red following 14 days in osteogenic culture media. Panel C, cells stained with Alcian blue following 21 days in chondrogenic culture. Black scale bars 100μm. D, Graphical representation of the number of adipocytes in each group. Error bars represent ± SEM. *P<0.05, Fetal vs MV and SB vs MV. n = 3 for each group, with all experiments performed in quadruplicate. Panel E depicts mRNA expression of VEGF and Panel F and G, HIF1α and BMP4 mRNA expression respectively. mRNA expression was assessed by real-time RT-PCR and then normalized to GAPDH mRNA levels. The results are the mean ± S.E of three separate experiments performed in triplicate. *P<0.05, Fetal vs MV.

L-MSCs were subjected to lineage differentiation assays following *in vitro* stimulation. Cells isolated from the SB and Fetal groups showed a robust adipogenic potential (Fig 2, Panel A). Cells from lungs exposed to MV demonstrated a lower adipogenic potential (Fig 2, Panel A and D). Similarly, L-MSCs from the Fetal and SB groups formed large aggregates/precipitates of calcium phosphate as revealed by the intense dark red stains; however, calcium phosphate deposits were much smaller and sparse in cells isolated from the MV group (Fig 2, Panel B). In contrast to the adipogenic and osteogenic differentiation, no difference in chondrogenic differentiation potential was seen among the three groups (Fig 2, Panel C).

Studies suggest that MSCs promote tissue repair via secretion of biologically active growth factors [22,23]. Vascular endothelial growth factor (VEGF) mRNA expression showed a significant reduction in L-MSCs from the MV group compared to levels detected in the Fetal group (Fig 2, panel E). Hypoxia inducible factor-1α (HIF-1α) mRNA abundance was decreased in L-MSCS isolated from kits in the MV group compared to L-MSCs from the Fetal group but did not reach statistical significance (p = 0.22, Fig 2, panel F). Bone morphogenetic protein (BMP) 4 mRNA expression was similar in the groups (Fig 2, panel G).

**Fig 3:**
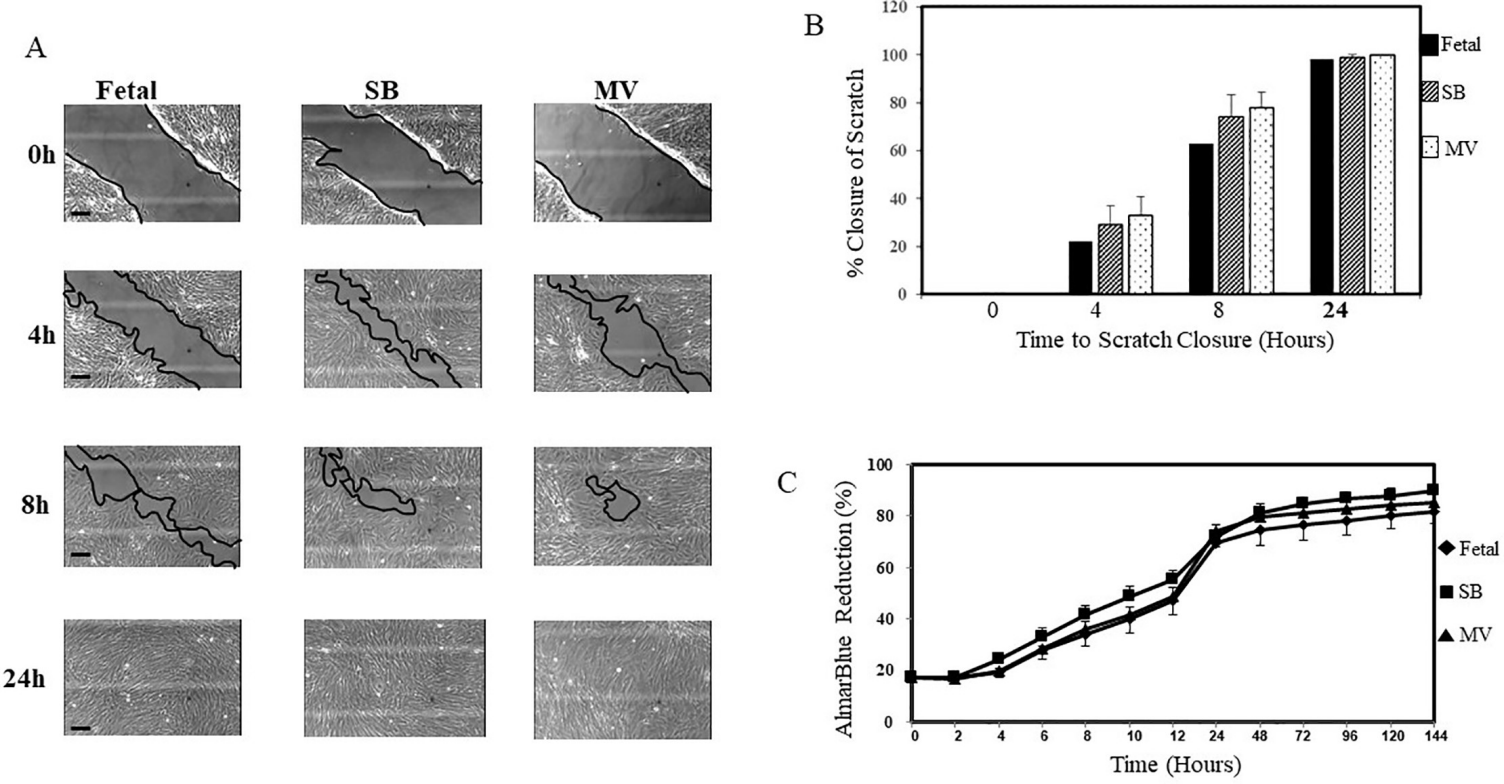
Scratch and proliferation assay. Panel A, phase contrast images of migration potentially L-MSCs isolated from each experimental group, respectively. Each row designates a specific time point, beginning at 0h of the assay when the gap was created till 24h. Black scale bars 100μm. Panel B, graphical representation of the percentage closure of the scratch at each time point for each of the three groups. Error bars represent ± SEM. Fetal = black box, SB = diagonal lines box, MV = dotted box. Experiments were performed in duplicate with six separate wells/experimental group. Panel C graphical representation of the proliferation rate for L-MSCs isolated from each experimental group using the AlmarBlue assay. Cells were plated in a 96-well plate and analyzed every 2h up to 144h. Each group exhibits the typical s-shaped curve seen with cell proliferation. n = 4 for each group with quadruplicate samples. Data is shown as ± SEM.

As shown in Fig 3 panels A and B, utilizing a scratch test assay, the migratory/wound healing properties of L-MSCs isolated from our three experimental groups were similar. Cell growth curves displayed a typical S-shaped curves (Fig 3, panel C). There were no significant differences in L-MSCs proliferation rates between the three groups with all L-MSCs reaching the plateau phase between 48 and 72h. Analysis was conducted using repeated measures ANOVA (p = 0.86).

### Transmission Electron Microscopy (TEM) ultrastructural analysis of rabbit L-MSCs

**Fig 4:**
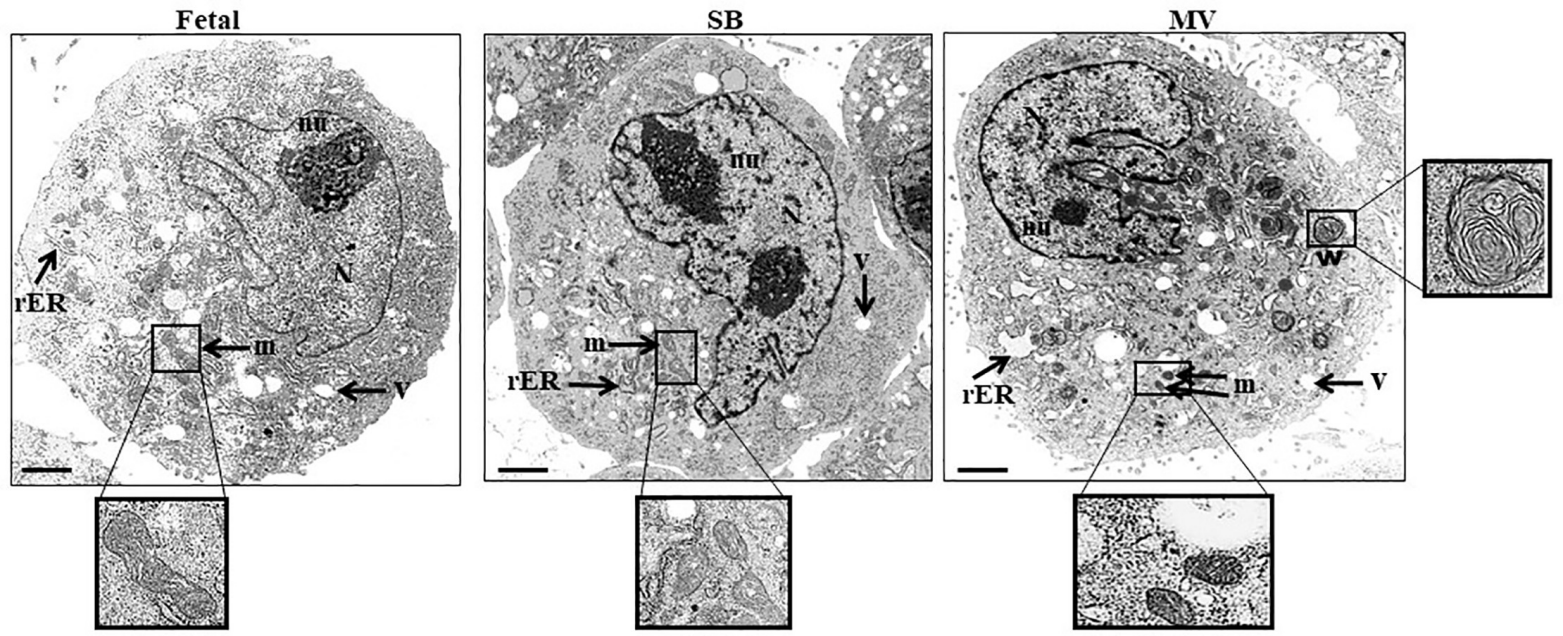
Ultrastructure characteristics of L-MSCs. Three separate samples of L-MSCs from each experimental group were analyzed and representative TEM images were obtained. L-MSCs have large, irregular shaped nucleus (N) with prominent nucleoli (nu). Mitochondria (m) appear tubular in the Fetal and SB groups, whereas they are small and rounded in the MV group (enlarged area in box). Whorls (w) are only present in the MV group. Black scale bars 2μm.

We assessed the effects of hyperoxia and mechanical ventilation on ultrastructural characteristics in L-MSCs with transmission electron microscopy (TEM). Representative TEM images of cultured L-MSCs are depicted in Fig 4. L-MSCs possessed a large, irregular shaped nucleus with one or two prominent nucleoli, numerous vacuoles (v), endoplasmic reticuli (ER), and mitochondria (m). The nuclei in fetal L-MSCs were predominantly euchromatic, whereas the nuclei in L-MSCs from the SB and MV groups exhibited heterochromatin margination (as evidenced by dark stained areas within the nucleus and nuclear envelope). The presence of whorls (indicated by the letter w), a sign of cellular stress, was only detected in L-MSCs isolated from kits in the MV group.

**Fig 5:**
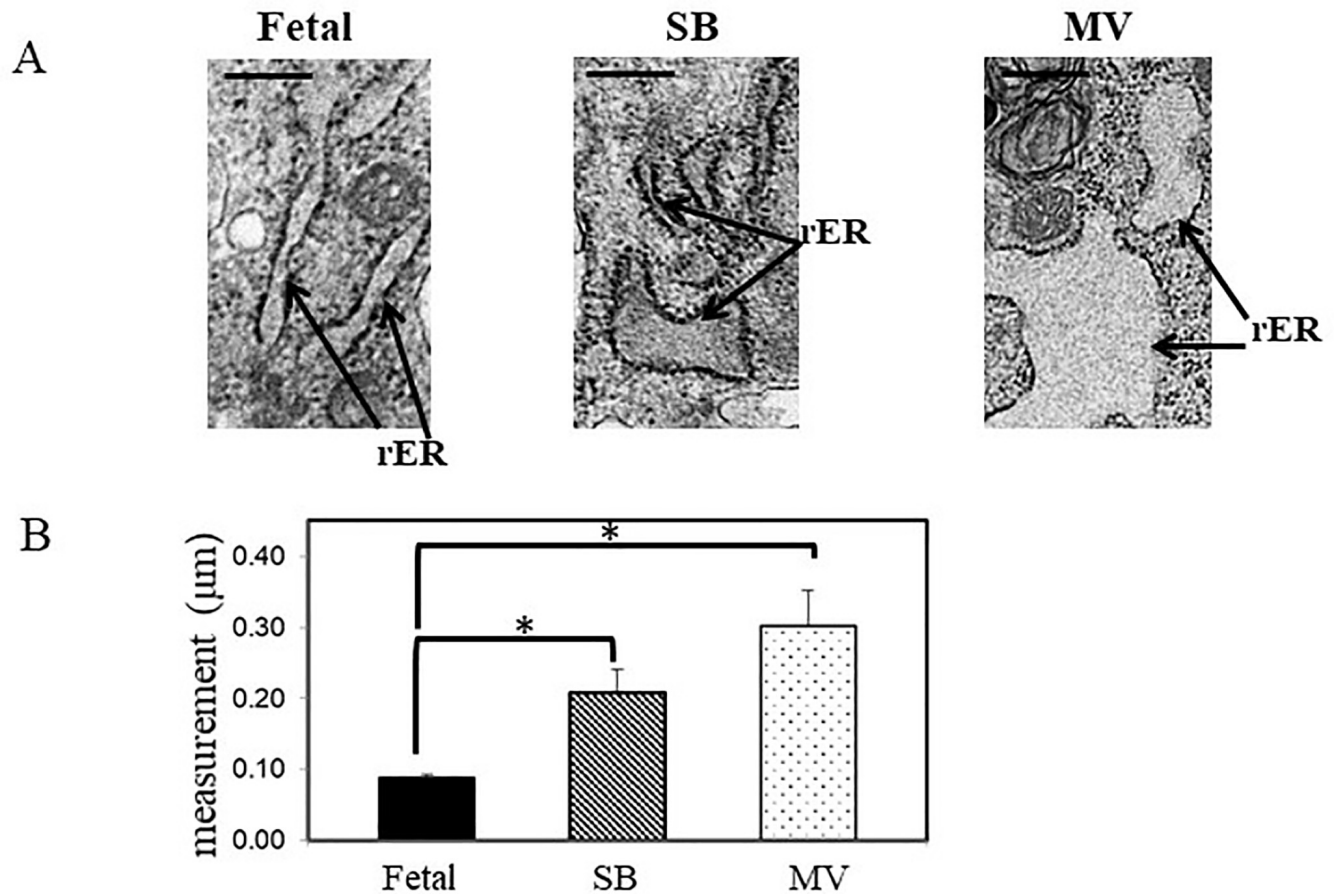
Dilation of endoplasmic reticulum (ER). Panel A, representative TEM images depicting progressive dilation of the ER in L-MSCs isolated from each of the experimental groups. Black scale bars 500nm. Panel B, quantitative analysis representing the degree of ER dilation. The results are the mean ± S.E of three experiments performed in triplicate. *P<0.05, Fetal vs MV and SB vs Fetal.

Fetal L-MSCs mitochondria appeared elongated and tubular in shape versus shorter mitochondria in the SB group. Furthermore, the mitochondria observed in L-MSCs isolated from the MV experimental group, were smaller and displayed a more rounded structure. Measurements were taken to achieve a quantitative estimate of the amount of dilation of the ER in L-MSCs. As depicted in Fig 5, panel A and B, L-MSCs from the SB and MV groups were significantly more dilated compared to narrower rER observed in the L-MSCs from the Fetal group.

### Effect of hyperoxia and mechanical ventilation on differential gene expression in rabbit L-MSCs

Next, we compared the differences in gene expression patterns that were induced in L-MSCs concomitant with routine preterm neonatal care by performing oligonucleotide microarray experiments. S2 Table depicts the overall number of genes that were differentially expressed (foldchange ≥1.5) in L-MSCs isolated from the three experimental groups. Selected top up- and down-regulated genes are detailed in Tables 2–4. S3–S5 Tables list all the differentially expressed genes based on the fold change with the respective gene symbol.

**Table 2:** Top 10 differentially upregulated genes and 5 downregulated genes in L-MSCs from SB versus fetal group (fold change ≥1.5, *P*<0.05).

UPREGULATED			
Gene Symbol	Name	Fold Change	P Value
*FRZB*	Frizzled-related protein	2.43	3.03E-05
*RGS5*	Regulator of G protein signaling 5	2.27	4.58E-02
*DAPL1*	Death associated protein like 1	2.02	7.81E-04
*ANO4*	Anoctamin 4	1.95	1.68E-03
*COCH*	Cochlin	1.93	2.13E-02
*HOXA11*	Homeobox A11	1.93	5.56E-02
*COL13A1*	Collagen type XIII alpha 1 chain	1.91	3.70E-03
*ITGB1BP2*	Integrin subunit beta 1 binding protein 2	1.91	7.14E-02
*NID2*	Nidogen 2	1.91	1.78E-03
*GRB14*	Growth factor receptor bound protein 14	1.91	9.62E-03
**DOWNREGULATED**			
*NTS*	Neurotensin	-2.80	1.57E-02
*NPY*	Neuropeptide Y	-2.01	3.60E-04
*DPT*	Dermatopontin	-1.75	1.94E-02
*MGST1*	Microsomal Glutathione S-Transferase 1	-1.60	5.18E-02
*RIOK1*	RIO kinase 1	-1.47	4.52E-03

**Table 3:** Top 10 differentially up/down-regulated genes in L-MSCs isolated from MV versus fetal group (fold change ≥1.5, *P*<0.05).

UPREGULATED			
Gene Symbol	Name	Fold Change	P Value
*POSTN*	Periostin	3.92	2.23E-03
*ITGB1BP2*	Integrin subunit beta 1 binding protein 2	3.30	8.26E-03
*RGS5*	Regulator of G protein signaling 5	3.12	1.50E-02
*MGP*	Matrix Gla protein	2.68	7.21E-03
*PBLD*	Phenazine biosynthesis like protein domain containing	2.65	1.75E-02
*IL17B*	Interleukin 17B	2.60	7.43E-03
*HOXA11*	Homeobox A11	2.59	2.11E-02
*ATP6V0D2*	ATPase, H+ transporting, lysosomal V0 subunit D2	2.41	2.10E-02
*ANO4*	anoctamin 4	2.30	8.45E-04
*COL13A1*	Collagen type XIII alpha 1 chain	2.26	1.93E-03
**DOWNREGULATED**			
*F3*	coagulation factor III, tissue factor	-4.57	1.16E-03
*RRM2*	ribonucleotide reductase regulatory subunit M2	-3.98	1.56E-04
*CCNB2*	cyclin B2	-3.78	9.24E-04
*MKI67*	marker of proliferation Ki-67	-3.71	9.39E-03
*NCAPH*	non-SMC condensin I complex subunit H	-3.67	1.33E-03
*SKA1*	spindle and kinetochore associated complex subunit 1	-3.66	4.20E-03
*POLE*	DNA polymerase epsilon, catalytic subunit	-3.57	1.91E-03
*AURKB*	aurora kinase B	-3.54	1.64E-03
*CDC20*	cell division cycle 20	-3.52	1.84E-03
*NEK2*	NIMA related kinase 2	-3.52	2.30E-03

**Table 4:** Differentially upregulated/downregulated genes in L-MSCs isolated from MV versus SB preterm rabbits (fold change ≥1.5, *P*<0.05).

**UPREGULATED**			
**Gene Symbol**	**Gene Name**	**Fold Change**	**P Value**
*ATP6V0D2*	ATPase, H+ transporting, lysosomal V0 subunit D2	2.60	1.03E-02
*POSTN*	Periostin	2.43	2.35E-02
*SERPINB9*	Serpin family B member 9 [*Homo sapiens*	1.96	8.80E-03
*IL17B*	interleukin 17B	1.71	4.18E-02
*MDGA1*	MAM domain containing glycosylphosphatidylinositol anchor 1	1.66	3.43E-02
*TUFT1*	Tuftelin 1	1.54	3.30E-02
*ITGB2*	Integrin subunit beta 2 [*Homo sapiens*	1.49	2.52E-03
**DOWNREGULATED**			
*F3*	Coagulation factor III, tissue factor	-3.26	6.88E-03
*CCNB2*	Cyclin B2	-3.24	1.74E-03
*SLC31A1*	Solute carrier family 31 member 1	-3.24	3.83E-04
*RRM2*	Ribonucleotide reductase regulatory subunit M2	-3.19	5.37E-04
*NCAPH*	Non-SMC condensin I complex subunit H	-3.15	2.43E-03
*CDC20*	cell division cycle 20	-2.97	3.65E-03
*MKI67*	Marker of proliferation Ki-67	-2.92	2.25E-02
*UBE2C*	Ubiquitin conjugating enzyme E2 C	-2.91	7.45E-03
*BUB1*	BUB1 mitotic checkpoint serine/threonine kinase	-2.91	2.95E-03
*SKA1*	Spindle and kinetochore associated complex subunit 1	-2.90	1.10E-02

**Fig 6:**
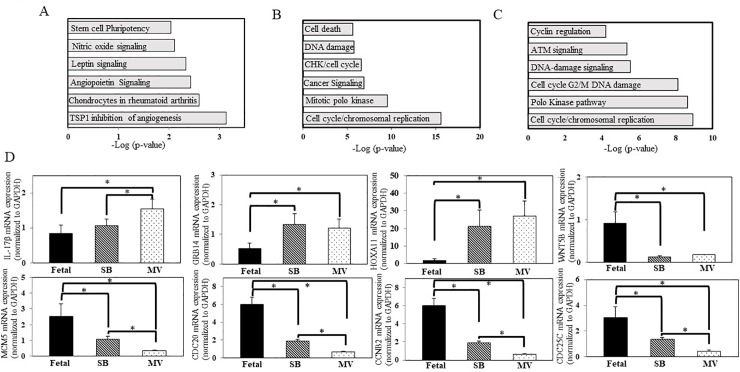
Canonical pathways and RNA expression of candidate genes altered following postnatal adaptation in L-MSCs. Panel A-C, Bar graphs depicting chief functional pathways represented by genes isolated from each experimental group. The genes included are those demonstrating ≥1.5-fold change in expression. N = 4 in each group; *P* < 0.05. (**A**) spontaneous breathing vs. fetal; (**B**) mechanical ventilation vs. fetal; (**C**) mechanical ventilation vs. spontaneous breathing. Panel D, mRNA expression of a series of candidate genes assessed by real-time RT-PCR following isolation of total RNA from cultured L-MSCs from each experimental group. Expression level change of all candidate genes was normalized to the housekeeping gene GAPDH. Quantitative RT-PCR results are presented as mean ± SE. *P<0.05, of three separate experiments performed in triplicate.

Differentially expressed genes from each of the comparison groups were further analyzed using Ingenuity Pathway Analysis (IPA) to identify the topmost canonical pathways associated with regulators, signaling pathways, or biological functions. Fig 6A–6C, are bar graphs depicting the chief canonical pathways represented by the genes differentially altered between groups. The main observed pathways that were altered following exposure to 50% O_2_ included regulation of angiogenesis. Exposure to mechanical ventilation with supplemental 50% O_2_ altered several pathways involved in the cell cycle control of chromosomal replication and cell division. We then validated gene expression results by quantitative RT-PCR. We choose 8 candidate genes that were significantly expressed and exhibited good correlation with the results from the microarray analysis, Fig 6, panel D.

## Discussion

Mesenchymal stromal cells (MSCs) therapy has emerged as a novel treatment modality for problematic neonatal lung disease such as BPD. Successful pilot studies in newborn hyperoxia-induced BPD rodent models confirmed the therapeutic potential of not only exogenous MSCs, but also MSC-free conditioned media and MSC-derived exosomes for the attenuation of lung tissue injury [24–26]. More recently, phase I trials in preterm babies at risk of developing BPD have successfully reported that administration of allogenic cord blood-derived MSCs was well tolerated at the time and devoid of respiratory and neurodevelopmental deficits at the 2-year follow-up evaluation [27]. While there is emerging literature on the use of exogenous MSCs and its derivatives to alleviate neonatal lung disease, there is a gap in knowledge on the properties/characteristics of resident/endogenous lung MSCs (L-MSCs). This cell population drives lung tissue growth, repair, and regeneration and understanding how these properties are altered during premature extrauterine postnatal adaptation is important. To this end, we investigated whether exposure to hyperoxia and mechanical ventilation with supplemental oxygen for 4h modified the biological characteristics of resident MSCs isolated from the preterm rabbit lung.

Exposure of kits to either hyperoxia or mechanical ventilation with supplemental oxygen for 4h did not affect the proliferative, migratory, and chondrogenic capacity of adherent L-MSCs. However, L-MSCs from MV kits formed significantly less colonies and demonstrated a lower adipogenic and osteogenic capacity than L-MSCs from unventilated and SB kits.

The stem cell transcription factor SOX-2, a regulator of pluripotency, has been previously identified in rabbit bone marrow stromal cells [21]. In agreement with studies by Möbius and colleagues, we were able to detect SOX-2 expression in rabbit L-MSCs [28]. As postnatal adaptation, namely hyperoxia and mechanical ventilation with hyperoxia for 4h did not significantly diminish expression of SOX-2, at this point we speculate that some of the pluripotent properties of resident L-MSCs remain intact. Furthermore, we observed a decrease in CD81 expression in L-MSCs isolated from the MV group compared to L-MSCs isolated from kits exposed to hyperoxia (SB group). Interestingly, CD81 is also a well-described marker for exosomes, which are a component of the MSC-derived secretome that promote the repair of surrounding injured cells and tissue [26,29].

Ultrastructural TEM analysis indicated that L-MSCs isolated from each experimental group possessed several vacuoles, large, irregularly shaped euchromatic nucleus with prominent nucleoli [30,31]. Heterochromatin margination was predominant in L-MSCs isolated from the SB and MV experimental groups and may be attributed to environmental or surrounding changes exceeding the capacity of the cell to maintain normal homeostatic function. In most instances, if the insult is short and not profound, the cells may revert to normal function. Heterochromatin has also been reported to exert a protective role in maintaining the stability of chromosomal regions, which otherwise would be susceptible to functional and structural damage [32]. The prevalence of heterochromatin margination associated with a high oxygen concentration was previously described in alveolar type I and type II cells isolated from neonatal rat lungs following exposure to 85% hyperoxia for 14 days [33]. Vacuoles containing concentric whorl-like structures were only present in the cytoplasm of L-MSCs isolated from kits assigned to the MV with hyperoxia group. It has been previously reported that the formation of cytoplasmic whorls occurs because of cell or membrane damage in response to cellular stress [34].

Prolonged mechanical ventilation with hyperoxia increases the cellular accumulation of reactive oxygen species [ROS, 9,35,36]. Mitochondria and endoplasmic reticulum (ER) are highly sensitive to oxygen concentrations and if overwhelmed, oxidative stress can eventually cause organelle dysfunction resulting in tissue injury [37,38]. Changes in the oxidative state of a cell are reflected by the congruent shape of mitochondria [39]. Environments with high reactive oxygen species cause mitochondria to change from a long-tubular shape to a rounded, punctate structure [37,38]. Here we show that mitochondria in L-MSCs isolated from the fetal experimental group exhibited a tubular-like appearance, in contrast to more rounded and smaller structures identified in L-MSCs isolated from the SB and MV experimental groups. In conjunction, we also report here that the ER was significantly swollen in L-MSCs previously exposed to hyperoxia; distension of the ER was markedly pronounced in L-MSCs exposed to mechanical ventilation with hyperoxia. A study by Teng and colleagues demonstrated that hyperoxia-induced dilation of the ER is associated with elevated ER stress and affects the coupling interactions between the ER and mitochondria [38]. It is of consequence to note here that one of the mechanisms by which MSCs promote cellular repair is by the transfer of functional mitochondria enclosed in microvesicles to injured cells [40]. It is indeed detrimental for MSCs themselves to have functionally compromised mitochondria as this may indeed lessen their reparative capacity.

L-MSCs express a variety of transcription and growth factors including vascular endothelial growth factor (VEGF), that not only promote tissue repair but also foster lung growth and development [7,41]. During normal development, the low oxygen tension regulates expression of VEGF in the fetal lung via hypoxia inducible factors [HIFs, 42]. *In vitro* studies by Möbius and colleagues reported that human fetal L-MSCs cultured in 60% O_2_ for 7 days secreted minimal amounts of VEGF compared to cells cultured in a hypoxic environment [28]. Similarly, we report here that VEGF mRNA expression was significantly decreased in L-MSCs isolated from the MV with hyperoxia experimental group compared to the fetal group. Although VEGF mRNA expression was lower in L-MSCs isolated from the SB group it did not reach statistical significance. Possible reasoning for our findings could be due to short-term exposure of the lung-tissue resident MSCs to the high concentration of O_2_.

Microarray analysis was used to assess key changes in resident L-MSCs gene expression. Expression of IL-17β, a proinflammatory cytokine, increased in L-MSCs isolated from the SB and MV experimental groups. This cytokine is known to contribute to the pathogenesis of chronic inflammatory lung diseases including asthma and chronic obstructive pulmonary disease [43]. Similarly, L-MSCs from the SB and MV experimental groups had an increased expression of growth factor receptor-bound protein 14 (GRB14). In a placebo-controlled asthma intervention study, GRB14 was among the down-regulated genes explaining the effects of prednisolone on airway smooth muscle remodeling [44]. Homeobox protein A11 (HOXA11) and WNT5B are genes involved in the regulation, proliferation, and differentiation of developing tissues. A brief exposure of oxygen or oxygen with mechanical ventilation in preterm rabbit L-MSCs created an overexpression of HOXA11 and an under expression of WNT5B. An imbalance in the expression of these genes likely leads to an impairment in normal lung development [45,46].

A recent study by Shivanna and colleagues observed that following exposure of human fetal lung cells to hyperoxia, genes that were altered included those participating in the regulation of cell cycle and cell division [47]. In our study, CDC20 and cyclin B2 were downregulated in L-MSCs isolated from the SB and MV groups. Decreased gene expression in these cell cycle regulators may, in part, result in alveolar hypoplasia that is commonly seen in preterm newborns with BPD [2]. Taken together, our findings suggest that hyperoxia and/or mechanical ventilation interrupts endogenous L-MSCs function. Furthermore, an arrest of the endogenous L-MSCs cell cycle processes may in turn contribute to epigenetic changes resulting in the decreased alveolar growth observed in BPD.

Corroborating the *in vitro* endogenous human fetal lung MSC findings reported by Möbius et al [28], supplemental oxygen with/without ventilation: 1) upregulated the production of fibrotic transcripts (POSTN, COL13A1); 2) decreased the secretion of angiogenic factors (e.g. VEGF); and 3) altered L-MSC surface markers and reduced colony forming ability. Collectively, these changes may contribute to the decreased reparative or restorative effects of endogenous L-MSCs observed in animal studies of hyperoxia-induced BPD [24,25]. Thus, it is possible that the therapeutic effects of exogenously delivered MSCs may be due to compensatory mechanisms that were lost in the injured endogenous lung MSCs.

## Conclusion

Short term exposure to hyperoxia or hyperoxia with ventilation induces anatomic, functional, and genomic changes in preterm rabbit resident/endogenous L-MSCs. These alterations may negatively impact their reparative/regenerative potential. It is currently unknown as to whether endogenous L-MSCs recover and regain their biological properties after cessation of postnatal interventions. Additional studies focusing on different pre- and postnatal insults on endogenous L-MSCs will further our understanding of the role these cells play during lung development. Recent studies have clearly demonstrated the therapeutic efficacy of exogenously applied MSC and/or their secretome, but the interaction between exogenously administered MSCs and their effects on endogenous L-MSCs is unclear [28–30]. Understanding these studies is immensely valuable and may provide new mechanistic pathways, and therefore targets for future therapies.

## Supporting information

S1 Table RNA primers. (DOC)

S2 Table Number of genes differentially expressed in L-MSCs from the experimental groups. (DOC)

S3 Table Differentially upregulated genes and downregulated genes in L-MSCs isolated from SB vs. Fetal (fold change ≥1.5, *P*<0.05). (DOC)

S4 Table Differentially upregulated genes and downregulated genes in L-MSCs isolated from MV vs. Fetal (fold change ≥1.5, *P*<0.05). (DOC)

S5 Table Differentially expressed genes in L-MSCs isolated from MV vs. SB (fold change ≥1.5, *P*<0.05). (DOC)

S1 Fig (JPG)

S2 Fig (JPG)
