# Supplementary material for: Oxygen and mechanical ventilation impede the functional properties of resident lung mesenchymal stromal cells
Source: PLoS One. 2020 Mar 6;15(3):e0229521. doi: 10.1371/journal.pone.0229521 (PMC7064315; doi:10.1371/journal.pone.0229521)
Supplement: S1 Table — (DOC) [file pone.0229521.s001.doc]

**Table S1.** RNA primers.

| Primer | Forward | Reverse |
| --- | --- | --- |
| SOX-2 | 5-CGCGGCGGAAAACCA-3 | 5-CCTCCGGGAAGCGTGTACT-3 |
| NANOG | 5-CCCAGGTGCCTCTTACAGACA-3 | 5-GAGTGGGCAAGTCCATGGA-3 |
| GRB14 | 5- AGTGGCTGCAGTTCTCAGAGTATAT-3 | 5- GAGAAGTCCATTGCTACCAGAGAAT-3 |
| IL-17 | 5-GGCTAAGCGAGAACAGTGCTA-3 | 5-GGATGCAGAGGAGCAATTATAGCT-3 |
| WNT5B | 5- TCATGAACCTGCAGAACAACGA-3 | 5- GGCTACGTCTGCCATCTTGTA-3 |
| MCM5 | 5- TCGCCTGCCTCCTGTTTG-3 | 5- GCCTCGCCGTGTGAGT-3 |
| CDC20 | 5-CAGCGCCGGCAGAAC-3 | 5- GGTGGTCTGTCCCTTGGAATT-3 |
| CCNB2 | 5-GACCCTTGCCCTTACACTTCTTAA-3 | 5- CAATGAGAGTCAGCTCCATCAAGTA-3 |
| CDC25C | 5-GCAGAAAGAAAGATGCAGTGTGTAC-3 | 5- GCCACTGGGAAGGCTTCAAA-3 |
