# Supplementary material for: Oxygen and mechanical ventilation impede the functional properties of resident lung mesenchymal stromal cells
Source: PLoS One. 2020 Mar 6;15(3):e0229521. doi: 10.1371/journal.pone.0229521 (PMC7064315; doi:10.1371/journal.pone.0229521)
Supplement: S2 Table — (DOC) [file pone.0229521.s002.doc]

**Table S2. Number of genes differentially expressed in L-MSCs from the experimental groups.**

| Gene Altered  (≥1.5-fold, *P*<0.05) | SB vs. Fetal | MV vs. Fetal | MV vs. SB |
| --- | --- | --- | --- |
| Upregulated | 16 | 40 | 6 |
| Downregulated | 5 | 155 | 92 |
