# Supplementary material for: Oxygen and mechanical ventilation impede the functional properties of resident lung mesenchymal stromal cells
Source: PLoS One. 2020 Mar 6;15(3):e0229521. doi: 10.1371/journal.pone.0229521 (PMC7064315; doi:10.1371/journal.pone.0229521)
Supplement: S3 Table — (DOC) [file pone.0229521.s003.doc]

**Table S3.** Differentially upregulated genes and downregulated genes in L-MSCs isolated from **SB vs. Fetal** (fold change ≥1.5, *P*<0.05).

| Gene Symbol | Fold Change | P Value |
| --- | --- | --- |
| FRZB | 2.426834351 | 3.03E-05 |
| RGS5 | 2.27498696 | 0.045797186 |
| DAPL1 | 2.019223793 | 0.000781318 |
| ANO4 | 1.953667864 | 0.001681579 |
| COCH | 1.933865973 | 0.02132982 |
| HOXA11 | 1.928537576 | 0.055627022 |
| COL13A1 | 1.914444539 | 0.003702906 |
| ITGB1BP2 | 1.912867851 | 0.071448774 |
| NID2 | 1.912115875 | 0.001780787 |
| GRB14 | 1.911414319 | 0.009618628 |
| ARHGAP25 | 1.77834835 | 0.024135707 |
| PRSS12 | 1.746335305 | 0.001289715 |
| COL4A4 | 1.712824288 | 0.006066737 |
| RNASE4 | 1.704608948 | 0.021132024 |
| MTUS2 | 1.67389762 | 0.025497596 |
| SPARCL1 | 1.56317 | 0.02943 |
| NTS | -2.80095911 | 0.01568658 |
| NPY | -2.009187836 | 0.000359904 |
| DPT | -1.751893062 | 0.019398643 |
| MGST1 | -1.60156 | 0.05181 |
| RIOK1 | -1.469740036 | 0.004521862 |
