# Supplementary material for: Oxygen and mechanical ventilation impede the functional properties of resident lung mesenchymal stromal cells
Source: PLoS One. 2020 Mar 6;15(3):e0229521. doi: 10.1371/journal.pone.0229521 (PMC7064315; doi:10.1371/journal.pone.0229521)
Supplement: S4 Table — (DOC) [file pone.0229521.s004.doc]

**Table S4.** Differentially upregulated genes and downregulated genes in L-MSCs isolated from **MV vs. Fetal** (fold change ≥1.5, *P*<0.05).

| Gene Symbol | Fold Change | P Value |
| --- | --- | --- |
| POSTN | 3.927488965 | 0.002236285 |
| ITGB1BP2 | 3.304749518 | 0.008262242 |
| RGS5 | 3.115199353 | 0.015009523 |
| MGP | 2.681525788 | 0.007213668 |
| PBLD | 2.645369503 | 0.017527798 |
| IL17B | 2.598299475 | 0.00742466 |
| HOXA11 | 2.593057188 | 0.021127648 |
| ATP6V0D2 | 2.411846103 | 0.021020126 |
| ANO4 | 2.300231582 | 0.000845089 |
| COL13A1 | 2.259710798 | 0.00193145 |
| IBSP | 2.25388757 | 0.048895318 |
| RNASE4 | 2.164703438 | 0.0086432 |
| ECM2 | 2.112450556 | 0.001652239 |
| MFGE8 | 2.102097892 | 0.016737905 |
| GRB14 | 2.041612274 | 0.009664933 |
| BCHE | 1.988328663 | 0.036953146 |
| FBXO32 | 1.894103773 | 0.012311175 |
| DAPL1 | 1.88344666 | 0.002052985 |
| POMC | 1.861116841 | 0.004390119 |
| HOXC8 | 1.809253698 | 0.001884039 |
| HTR2B | 1.796137385 | 0.008544052 |
| PRSS12 | 1.72906271 | 0.002189909 |
| RRM2B | 1.714914554 | 0.02124759 |
| CDKN2B | 1.70368649 | 0.014531171 |
| DPEP2 | 1.673100517 | 0.027367303 |
| CSRP2 | 1.661872247 | 0.004028615 |
| NOX4 | 1.660099563 | 0.010504742 |
| FAP | 1.658216993 | 0.024060835 |
| SYNPO2 | 1.656836177 | 0.049358429 |
| TGFB3 | 1.649397844 | 0.011849354 |
| ACYP1 | 1.646743656 | 0.003646717 |
| CD200 | 1.591174397 | 0.007009924 |
| PIK3IP1 | 1.589180531 | 0.007560785 |
| MYOZ2 | 1.563502674 | 0.005741219 |
| CYTIP | 1.561121415 | 0.026417685 |
| NAGLU | 1.554917775 | 0.002954897 |
| OR52Z1 | 1.535983067 | 0.022757105 |
| TIAM1 | 1.527785801 | 0.000897996 |
| GABBR1 | 1.510822711 | 0.01463577 |
| F3 | -4.574750378 | 0.001164617 |
| RRM2 | -3.981190852 | 0.000156233 |
| CCNB2 | -3.784218733 | 0.00092427 |
| MKI67 | -3.707763496 | 0.009387044 |
| NCAPH | -3.672786001 | 0.001325418 |
| SKA1 | -3.660164532 | 0.004196012 |
| POLE | -3.571712378 | 0.001911084 |
| AURKB | -3.543047615 | 0.001640812 |
| CDC20 | -3.520728467 | 0.001839897 |
| NEK2 | -3.517996624 | 0.002301587 |
| UBE2C | -3.510943696 | 0.003606314 |
| NTS | -3.480521756 | 0.006918537 |
| NEIL3 | -3.471092206 | 0.001878565 |
| MCM5 | -3.409815887 | 0.001991589 |
| IL16 | -3.265449934 | 0.009262957 |
| TK1 | -3.262758602 | 0.001595378 |
| MCM10 | -3.259673038 | 0.000237334 |
| CCNB1 | -3.239130835 | 0.001032364 |
| BUB1 | -3.218147325 | 0.00236506 |
| GINS1 | -3.177898506 | 0.000750412 |
| CDC6 | -3.17610652 | 0.001006078 |
| GTSE1 | -3.136050449 | 0.002504094 |
| LPL | -3.132597336 | 0.008018653 |
| MYBL2 | -3.111092252 | 0.003512907 |
| ASPM | -3.091048887 | 0.018895476 |
| MGST1 | -3.056516851 | 0.0025897 |
| CDC25C | -3.051991781 | 0.000712532 |
| CDCA2 | -3.047347029 | 0.000809175 |
| LMNB1 | -3.027313239 | 0.001453241 |
| PSAT1 | -3.024016051 | 0.009177286 |
| TCF19 | -2.917964489 | 0.002100339 |
| RAD54L | -2.890726559 | 0.001811268 |
| GINS4 | -2.888344246 | 0.000973781 |
| BLM | -2.859138747 | 0.001201486 |
| TTK | -2.835830088 | 0.015163 |
| KIF11 | -2.830329433 | 0.001356489 |
| KIF15 | -2.829479674 | 0.000848538 |
| CDK1 | -2.822575204 | 0.001531066 |
| MCM7 | -2.801212818 | 0.000926621 |
| SHCBP1 | -2.740618488 | 0.01346745 |
| BRCA1 | -2.733462179 | 0.001112506 |
| MELK | -2.71362348 | 0.001416158 |
| KIFC1 | -2.710981149 | 0.00127602 |
| CDCA8 | -2.696789484 | 0.003111012 |
| AURKA | -2.692186056 | 0.002446489 |
| NCAPG | -2.687827776 | 0.003985619 |
| NDC80 | -2.6847169 | 0.006760683 |
| CENPT | -2.684497727 | 0.001584999 |
| KIF23 | -2.673744624 | 0.000962146 |
| NCAPG2 | -2.652288989 | 0.000508672 |
| PDGFRA | -2.64711076 | 0.015660426 |
| STIL | -2.629825293 | 0.000590091 |
| POLE2 | -2.619899746 | 0.002323198 |
| PBK | -2.612895598 | 0.003840543 |
| MCM4 | -2.601293217 | 0.001402418 |
| CYP1A1 | -2.600685751 | 0.011904906 |
| TOP2A | -2.593788087 | 0.011428756 |
| CKAP2L | -2.559550299 | 0.002956909 |
| UBE2T | -2.549155344 | 0.000898505 |
| CENPP | -2.545254933 | 0.006652117 |
| CEP55 | -2.537311117 | 0.001264605 |
| ACKR3 | -2.533428984 | 0.00346765 |
| PSMC3IP | -2.510194054 | 0.003654196 |
| HIST1H1A | -2.505794171 | 0.002243867 |
| KRT17 | -2.502424677 | 0.012669812 |
| KNTC1 | -2.488298738 | 0.001105283 |
| ESPL1 | -2.434725355 | 0.00436011 |
| PKP2 | -2.432367679 | 0.019176504 |
| CDC25B | -2.39120349 | 0.002406541 |
| RAD51AP1 | -2.370254894 | 0.000837298 |
| CENPN | -2.337279728 | 0.002403258 |
| E2F1 | -2.311055958 | 0.006097386 |
| RAD51 | -2.292860104 | 0.003163722 |
| GSG2 | -2.275229397 | 0.016301653 |
| TF | -2.26723417 | 0.006839627 |
| NCAPD2 | -2.261046277 | 0.00316875 |
| FBXO5 | -2.219498722 | 0.00899595 |
| CENPS | -2.187006031 | 0.001167907 |
| INCENP | -2.184342788 | 0.007870834 |
| LIG1 | -2.166900462 | 0.002241182 |
| EZH2 | -2.158303823 | 0.00076481 |
| FIGNL1 | -2.102770901 | 0.003474866 |
| KRT8 | -2.094049684 | 0.002325363 |
| TBX3 | -2.086115487 | 0.001568021 |
| MCM3 | -2.080910712 | 0.002895313 |
| SGO1 | -2.073023413 | 0.001290813 |
| MAD2L1 | -2.06157541 | 0.001586228 |
| WNT5B | -2.055342719 | 0.001845814 |
| SVEP1 | -2.054164844 | 0.044776028 |
| SLC31A1 | -2.052544236 | 0.012040133 |
| NEURL1 | -2.051694211 | 0.009897235 |
| KRT14 | -2.049636339 | 0.004070582 |
| TRAIP | -2.034608434 | 0.022411695 |
| KRT19 | -2.028254949 | 0.000828536 |
| MCM6 | -2.024665622 | 0.002206234 |
| PRIM1 | -2.020887382 | 0.002274895 |
| ADAMTS8 | -2.018990768 | 0.004389823 |
| PRR11 | -2.008897554 | 0.001502014 |
| NEBL | -2.002435177 | 0.000383464 |
| ABCC4 | -1.999977944 | 0.006697463 |
| CGNL1 | -1.987779849 | 5.18E-05 |
| KRT84 | -1.983259702 | 0.001962013 |
| TMEFF2 | -1.9744378 | 0.013386918 |
| NUSAP1 | -1.969788717 | 0.011575075 |
| CPXM1 | -1.959195556 | 0.018755789 |
| KRT4 | -1.954382623 | 0.001197091 |
| FAM111A | -1.948580197 | 0.000408663 |
| TPX2 | -1.92658269 | 0.001157608 |
| CDCA5 | -1.919502235 | 0.033691824 |
| DIAPH3 | -1.918906026 | 0.000198974 |
| FOXM1 | -1.872710702 | 0.007821552 |
| BCAT1 | -1.871011087 | 0.008653773 |
| WDHD1 | -1.869419293 | 0.001082553 |
| PLK4 | -1.843984494 | 0.012782579 |
| ADAMTS1 | -1.808700764 | 0.029708256 |
| MTBP | -1.80716151 | 0.009624774 |
| DTL | -1.807136786 | 0.004576731 |
| CDKN2C | -1.80105834 | 0.011084672 |
| KRT16 | -1.790385063 | 0.001559504 |
| LRR1 | -1.790296842 | 0.001538122 |
| PFAS | -1.770719743 | 0.004455264 |
| PMF1 | -1.760805528 | 0.007699614 |
| CA9 | -1.756208492 | 0.011243663 |
| AVP | -1.710209276 | 0.03299717 |
| RFC5 | -1.707696392 | 0.005985099 |
| IGFBP4 | -1.691153528 | 0.048652404 |
| HIPK1 | -1.668180201 | 0.006329805 |
| EFEMP1 | -1.664304999 | 0.039163582 |
| DSN1 | -1.6636112 | 0.01216436 |
| FOXF1 | -1.653929121 | 0.005214078 |
| COL5A3 | -1.645681431 | 0.03449937 |
| H2AFZ | -1.640903719 | 0.004528928 |
| SMC2 | -1.636392482 | 0.03397306 |
| NFIB | -1.63557548 | 0.017589082 |
| LGALS3 | -1.613942586 | 0.034580123 |
| GMNN | -1.612472896 | 0.001289826 |
| NUP62CL | -1.597996364 | 0.001300305 |
| CHAF1B | -1.58805427 | 0.008604782 |
| DCTPP1 | -1.584500249 | 0.012561509 |
| ADA | -1.567062448 | 0.003469 |
| POLA2 | -1.563516223 | 0.004097344 |
| NDC1 | -1.562820933 | 0.007016668 |
| SPDL1 | -1.558433571 | 0.005777184 |
| ANLN | -1.539847968 | 0.016342907 |
| PID1 | -1.538492525 | 0.013882286 |
| DTYMK | -1.531453767 | 0.013590379 |
| CCDC77 | -1.529305854 | 0.001225436 |
| GPR19 | -1.5276603 | 0.008588903 |
| MUTYH | -1.526056438 | 0.012899354 |
| NSUN3 | -1.522546746 | 0.046723076 |
| REEP4 | -1.519683066 | 0.009741075 |
| OIP5 | -1.511886854 | 0.037365819 |
| FANCM | -1.50683602 | 0.025518088 |
