# Supplementary material for: Oxygen and mechanical ventilation impede the functional properties of resident lung mesenchymal stromal cells
Source: PLoS One. 2020 Mar 6;15(3):e0229521. doi: 10.1371/journal.pone.0229521 (PMC7064315; doi:10.1371/journal.pone.0229521)
Supplement: S5 Table — (DOC) [file pone.0229521.s005.doc]

**Table S5.** Differentially expressed genes in L-MSCs isolated from **MV vs. SB** (fold change ≥1.5, *P*<0.05).

| Gene Symbol | Fold Change | P Value |
| --- | --- | --- |
| ATP6V0D2 | 2.595192322 | 0.010266242 |
| POSTN | 2.429216248 | 0.023475662 |
| SERPINB9 | 1.963633235 | 0.008797344 |
| IL17B | 1.705591288 | 0.041801892 |
| MDGA1 | 1.658739246 | 0.034334801 |
| ESM1 | 1.553770461 | 0.06902155 |
| TUFT1 | 1.536221383 | 0.032999063 |
| ITGB2 | 1.48968473 | 0.002516474 |
| F3 | -3.255070618 | 0.00688445 |
| CCNB2 | -3.238553881 | 0.001736344 |
| SLC31A1 | -3.238179288 | 0.00038298 |
| RRM2 | -3.187185713 | 0.000536565 |
| NCAPH | -3.145014394 | 0.002429304 |
| CDC20 | -2.968301967 | 0.003650601 |
| MKI67 | -2.92346606 | 0.022525182 |
| UBE2C | -2.913056964 | 0.007452716 |
| BUB1 | -2.910338673 | 0.002954013 |
| SKA1 | -2.902953922 | 0.010963297 |
| POLE | -2.891171653 | 0.004869439 |
| NEIL3 | -2.885896388 | 0.004063208 |
| AURKB | -2.881673433 | 0.004122061 |
| NEK2 | -2.864878725 | 0.005547673 |
| IL16 | -2.747774404 | 0.016224229 |
| CDC25C | -2.729294246 | 0.000991666 |
| MYBL2 | -2.724631631 | 0.005287903 |
| TK1 | -2.707124672 | 0.003543594 |
| LMNB1 | -2.663808491 | 0.00221059 |
| CYP1A1 | -2.61735744 | 0.007953706 |
| MCM5 | -2.606482392 | 0.00710023 |
| CCNB1 | -2.557087243 | 0.00328809 |
| CDCA2 | -2.526919311 | 0.001903548 |
| GTSE1 | -2.462312317 | 0.007408308 |
| GINS1 | -2.455080435 | 0.002852427 |
| MCM10 | -2.447969016 | 0.001253758 |
| TCF19 | -2.445601783 | 0.004281658 |
| KIF15 | -2.436345229 | 0.001534971 |
| CENPP | -2.425189424 | 0.00596641 |
| RAD54L | -2.318115437 | 0.004914928 |
| TTK | -2.283877455 | 0.030781379 |
| CDK1 | -2.224792434 | 0.004706277 |
| SHCBP1 | -2.222770401 | 0.026938863 |
| GINS4 | -2.215775059 | 0.003748698 |
| KIF23 | -2.188264328 | 0.002437975 |
| MELK | -2.186160092 | 0.003832658 |
| CENPT | -2.161784909 | 0.004251286 |
| FBLN2 | -2.160922908 | 0.015727734 |
| STIL | -2.15596367 | 0.001538516 |
| NCAPG2 | -2.136786025 | 0.001516146 |
| BLM | -2.128192917 | 0.005408634 |
| CKAP2L | -2.120484387 | 0.006279636 |
| BRCA1 | -2.119416543 | 0.003937546 |
| CDCA8 | -2.11193299 | 0.009073128 |
| KIF11 | -2.102411681 | 0.006065811 |
| LRRC17 | -2.101840596 | 0.019034135 |
| TF | -2.085521182 | 0.007536832 |
| KIFC1 | -2.071573747 | 0.004855344 |
| AURKA | -2.051593548 | 0.008607999 |
| ADAMTS8 | -2.047444192 | 0.002619148 |
| NCAPG | -2.044269598 | 0.013115981 |
| LPL | -2.011682124 | 0.048071654 |
| MCM7 | -2.007327498 | 0.005473013 |
| MCM4 | -1.999493327 | 0.005082389 |
| KNTC1 | -1.996328493 | 0.003149893 |
| CDC6 | -1.970158289 | 0.01313676 |
| GSG2 | -1.966931683 | 0.023861083 |
| KRT19 | -1.962088069 | 0.000645462 |
| PSMC3IP | -1.931364778 | 0.011468205 |
| POLE2 | -1.925240436 | 0.010089287 |
| THBS3 | -1.911291269 | 0.021926422 |
| NDC80 | -1.898495599 | 0.028472464 |
| CEP55 | -1.873541218 | 0.005925041 |
| UBE2T | -1.865833369 | 0.004689422 |
| PBK | -1.851228462 | 0.018244369 |
| CENPN | -1.845456865 | 0.006919457 |
| RAD51AP1 | -1.821820628 | 0.00324582 |
| CDC25B | -1.820690749 | 0.008529234 |
| TOP2A | -1.806736974 | 0.044947292 |
| ADAMTS1 | -1.772961189 | 0.024044741 |
| NCAPD2 | -1.762077701 | 0.009471256 |
| CDKN2C | -1.759068085 | 0.008739904 |
| NEBL | -1.742418503 | 0.000666356 |
| TRAIP | -1.724400098 | 0.034824185 |
| PRR11 | -1.650456464 | 0.0035704 |
| E2F1 | -1.648673485 | 0.025280203 |
| CENPS | -1.646982773 | 0.004919424 |
| RAD51 | -1.607732968 | 0.016516971 |
| KRT8 | -1.596827843 | 0.00821473 |
| INCENP | -1.57758944 | 0.029143146 |
| NUSAP1 | -1.567264263 | 0.025804196 |
| ESPL1 | -1.566584961 | 0.03130681 |
| MCM6 | -1.558482682 | 0.007444332 |
| TMEFF2 | -1.551409078 | 0.030849589 |
| HIST1H1A | -1.54819653 | 0.023649775 |
| FBXO5 | -1.542928054 | 0.037977445 |
| COQ5 | -1.535679471 | 0.021789739 |
| NSUN3 | -1.519084502 | 0.035987403 |
| PRIM1 | -1.508106565 | 0.009105907 |
| TPX2 | -1.501807793 | 0.003942445 |
