# Supplementary figures and images for: Oxygen and mechanical ventilation impede the functional properties of resident lung mesenchymal stromal cells
Source: PLoS One. 2020 Mar 6;15(3):e0229521. doi: 10.1371/journal.pone.0229521 (PMC7064315; doi:10.1371/journal.pone.0229521)

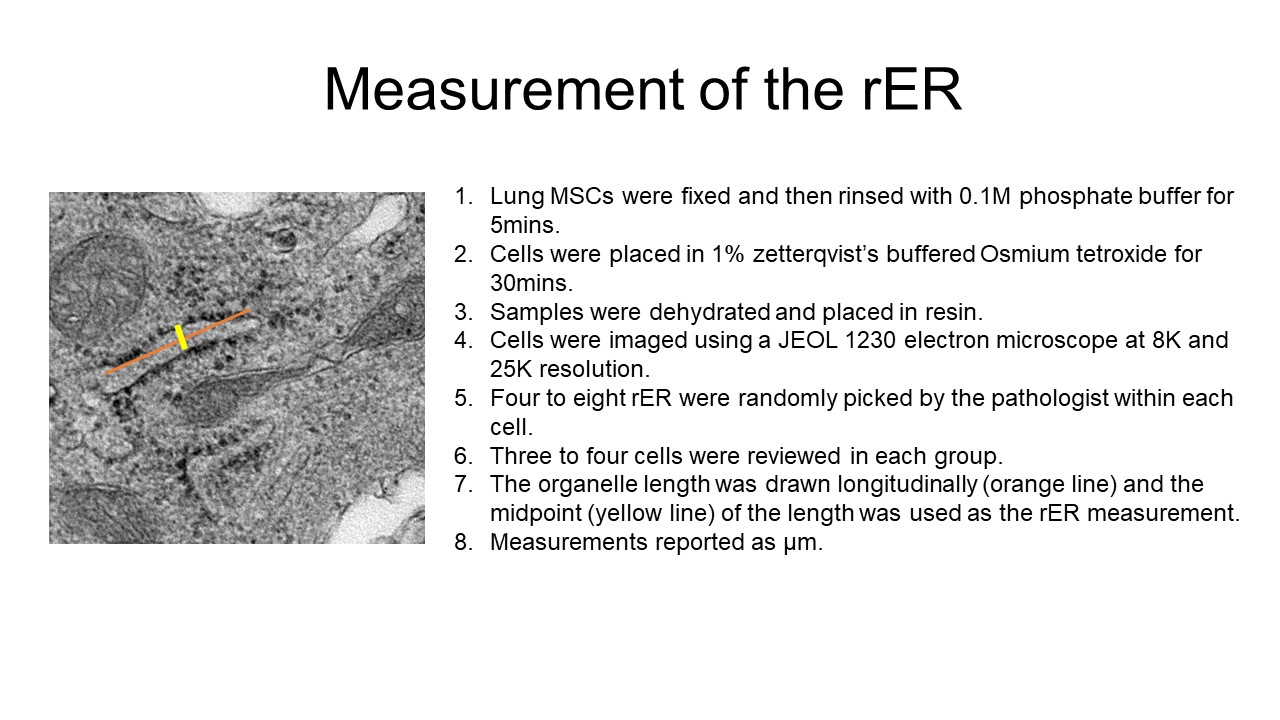

Supplement: S1 Fig — (JPG) [file pone.0229521.s006.jpg]

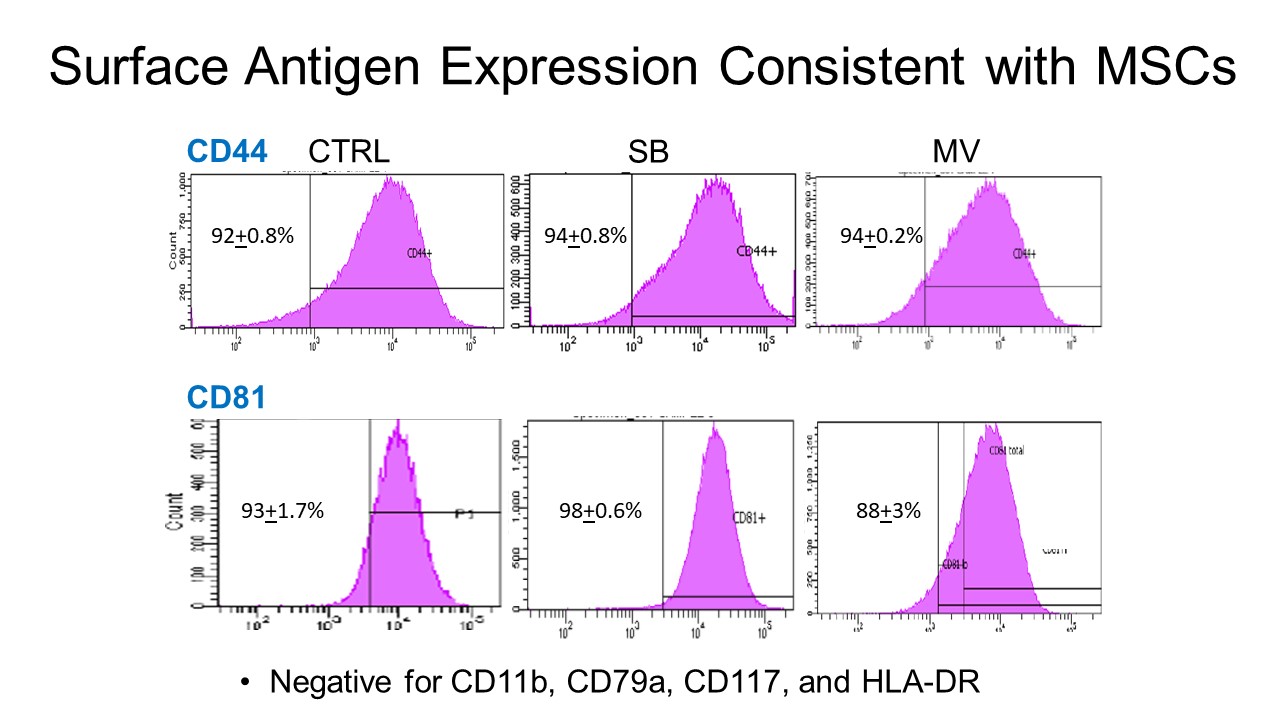

Supplement: S2 Fig — (JPG) [file pone.0229521.s007.jpg]
